# Salt-Affected Rocket Plants as a Possible Source of Glucosinolates

**DOI:** 10.3390/ijms24065510

**Published:** 2023-03-14

**Authors:** Emilio Corti, Sara Falsini, Cristina Gonnelli, Giuseppe Pieraccini, Besiana Nako, Alessio Papini

**Affiliations:** 1 Department of Biology, University of Florence, Via Micheli, 1-3, 50121 Florence, Italy; 2 Mass Spectrometry Centre, University of Florence, Viale Pieraccini, 6, 50139 Florence, Italy; 3 CSET—Tropical Herbarium, University of Florence, Via La Pira, 4, 50121 Florence, Italy

**Keywords:** *Eruca sativa*, glucosinolates, salinity, glucoerucin, glucosativin, nutraceuticals

## Abstract

Soil salinity can have various negative consequences on agricultural products, from their quality and production to their aesthetic traits. In this work, the possibility to use salt-affected vegetables, that otherwise would be discarded, as a source of nutraceuticals was explored. To this aim, rocket plants, a vegetable featuring bioactive compounds such as glucosinolates, were exposed to increasing NaCl concentrations in hydroponics and analysed for their content in bioactive compounds. Salt levels higher than 68 mM produced rocket plants that did not comply with European Union regulations and would therefore be considered a waste product. Anyway, our findings, obtained by Liquid Chromatography-High Resolution Mass Spectrometry, demonstrated a significant increase in glucosinolates levels in such salt-affected plants. opening the opportunity for a second life of these market discarded products to be recycled as glucosinolates source. Furthermore, an optimal situation was found at NaCl 34 mM in which not only were the aesthetic traits of rocket plants not affected, but also the plants revealed a significant enrichment in glucosinolates. This can be considered an advantageous situation in which the resulting vegetables still appealed to the market and showed improved nutraceutical aspects.

## 1. Introduction

Soil salinisation is one of the main issues affecting agriculture [1,2]. Every year, arable lands affected by saline increase by 10%, and, by 2050, 50% of the world’s arable lands are expected to be salinised [3]. Salt stress reduces the productivity and quality of cultivated plants, causing losses of 20–50%, since most crops are sensitive to salinity [4].

The impacts of salt stress on plants are several and affect various aspects, such as metabolic pathways, physiological processes, and morphological traits [5]. Thus, exposure to salinity results in a series of impairments that also modify the physical appearance of plants [6,7,8,9,10]. Visually, salt stress causes damage on the leaves, such as browning, discolouration, reduced area, and necrosis, which leads to a loss of aesthetic traits in cultivated plants [11]. Specifically, vegetable products were classified into categories by the EU’s Common Market Organisation (CMO) depending on their outer appearance. In ascending order of aesthetic features, there are Class II, Class I, and Extra Class, ranging from slight damage to impeccable traits [12]. From agricultural and trade perspectives, this leads to the frequent discarding of salt-affected vegetables due to their reduced commercial quality, since the final product does not fit with the standards that appeal to the final consumers [13,14].

On the other hand, abiotic stress conditions and particularly salinisation activate a precise cellular response that could result in the biosynthesis of specific secondary plant metabolites of possible interest for other purposes [15]. Orsini et al. [16] stated that, to cope with abiotic stress, vegetables synthesise and accumulate bioactive metabolites, such as ascorbate, glucosinolates, phenols, and/or carotenoids. For example, focusing on salt stress, salinity enhanced lycopene, vitamin C, and total phenolic concentration in tomato plants [17]. Plant species belonging to Brassicaceae are a natural source of glucosinolates, and *Eruca sativa* showed variation in glucosinolate concentration on the basis of genetic variability, as in the case of *Eruca sativa* cultivar Nemat [18].

In particular, salt stress enhanced the synthesis of glucosinolates, the characteristic compounds of the Brassicaceae family [19]. Guo et al. [20] observed an increase in glucoraphanin and sulforaphane in broccoli sprouts cultivated with 160 mM NaCl treatment; in radish sprouts [21] and *Arabidopsis* seedlings [22] grown under 100 mM NaCl treatment, there was an increase in total glucosinolate concentration. Moreover, Sarker and Oba (2018) [23] showed an increase in glucosinolate levels in kale, white cabbage, and Chinese cabbage cultivated with 50 and 100 mM NaCl treatments.

Glucosinolates are sulphur compounds derived from amino acids, showing a core structure formed by a β-D-glucopyranose residue linked to a (Z)-N-hydroximino sulphate ester via a sulphur atom and a variable side chain [24]. These molecules are classified as aliphatic, indolic, or aromatic depending on the feature of the initial amino acid [25]. In recent years, these bioactive compounds have been greatly relevant due to their beneficial effects on human health, showing antioxidant, anti-inflammatory, and antitumoral properties [26]. For instance, Steinbrecher et al. [27] showed a correlation between dietary glucosinolate intake and a reduction in prostate cancer insurgence. Moreover, positive effects on a reduction in several cancers such as lung [28], colon [29,30], bladder [31], and possibly breast [32], were reported for glucosinolates and their degradation compounds.

Brassicaceae plants are a potential source of glucosinolates to be used for medical purposes [33],and this use may be advantageous if cultivated in saline soils that would otherwise be unexploited. *Eruca sativa* Miller, commonly named arugula or rocket, is a native crop plant to the Mediterranean area belonging to the Brassicaceae family that currently has noteworthy relevance in the salad vegetable market [34,35]. This plant is also important because of its application to food conservation and innovative sustainable nanodrugs, given the remarkable biological significance of its phytoactive compounds [36,37,38]. Rocket is largely cultivated in various countries around the world, especially in the Mediterranean area [39]. In this region, 25% of irrigated cropland is affected by salinisation, and this percentage is expected to increase with climate change [40,41], thus also progressively impairing rocket production. In this context, several studies showed the negative effect of salt on *Eruca sativa* plant development [42,43,44]. Kusvuran and Ellialtioglu [45] found a reduction in glucosinolate amount in *E. sativa* plants grown in pots for 41 days below 80 mM NaCl. Furthermore, Petretto et al. (2019) [46] showed that salinity induced a decrease in isothiocyanates in rocket plants cultivated in saline soil substrates.

In light of the above, the aim of this study is to evaluate the variation in glucosinolate content in *Eruca sativa* Mill. plants grown under different salt levels, hypothesising that salt may affect the production of bioactive compounds as described in previously cited studies on Brassicaceae, although the plants may have low visual and aesthetic market values. Moreover, if saline conditions would enrich the plants in bioactive compounds without impairing the morphology, a win–win situation could provide a valuable nutraceutical product that is still appealing to consumers.

## 2. Results

### 2.1. Effect of Salt Stress on Leaf Area

**Figure 1:**
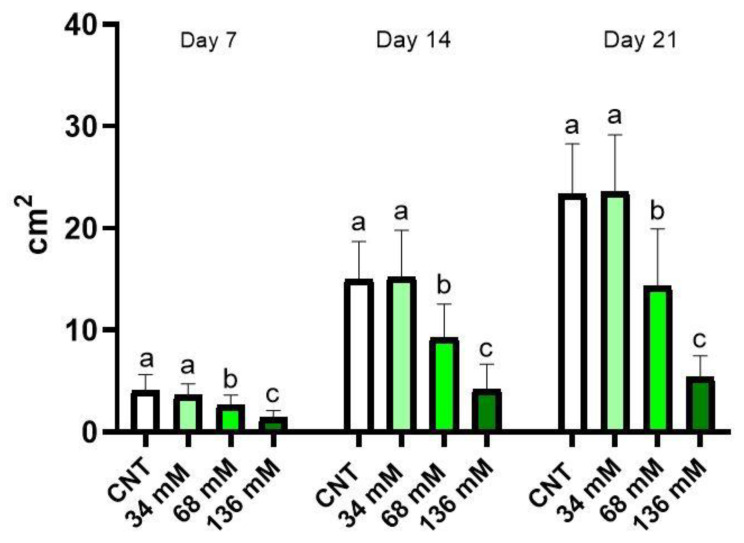
Aerial part surface of *E. sativa* plants grown under different saline conditions: Control (CNT), and 34, 68, and 136 mM NaCl. The graph shows the parameter measured after 7, 14, and 21 days from the beginning of the treatment. Different letters mean a statistical difference between the groups (*p* < 0.05).

Figure 1 shows the leaf surface of the control and salt-treated plants measured weekly for 3 weeks. After 7 days, a significant reduction IN this parameter in plants cultivated at the two highest salt concentrations (68 and 136 mM) was detectable if compared with the control and NaCl 34 mM groupS. The highest reduction in shoot surface was observed in plants subjected to the salt treatment of NaCl 136 mM. No significant differences were found between the control and the group grown with the lowest saline level (34 mM). The same trend was also observed after 2 and 3 weeks of treatment.

### 2.2. Damage Analysis

**Figure 2:**
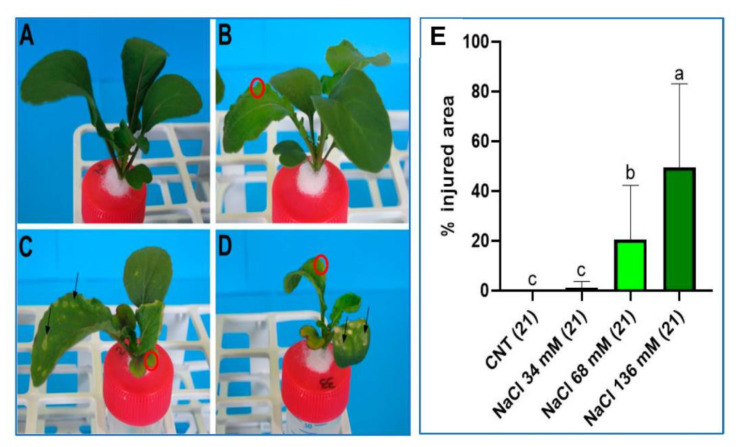
Images of the plants of the (**A**) control, (**B**) 34 mM NaCl, (**C**) 68 mM NaCl, and (**D**) 136 mM NaCl groups after 21 days of treatment. Black arrows show necrotic areas. Red circles indicate examples of chlorosis-affected zones. (**E**) Percentage of the plant shoot area affected by salt injuries in all four groups after 21 days of treatment. Different letters indicate a significant difference between the groups (*p* < 0.05).

Figure 2 shows the effect of salt stress on the physical appearance of the plants after 21 days. Salt-induced chlorosis and necrosis were recorded on the cotyledons and the older leaves of the plants, especially for the samples under the two highest saline levels, as shown in Figure 2C,D, respectively. The necrotic areas showed a rounded shape affecting both the lamina and the edge of leaves and cotyledons (Figure 2C,D). The chlorosis mainly affected the leaf and cotyledon margins, displaying a yellow–green colour (Figure 2B–D). In visual observation, necrosis was often associated with chlorosis.

The percentage of the shoot area affected by damage increased with increasing saline level (Figure 2E). The plants cultivated at the highest salt concentration (136 mM) showed damage on 50% of the shoot, while plants grown at 68 mM NaCl concentration displayed damage on 20% of the shoot area. At the lowest salt level, plants showed injuries only in 1% of the total aerial surface.

**Figure 3:**
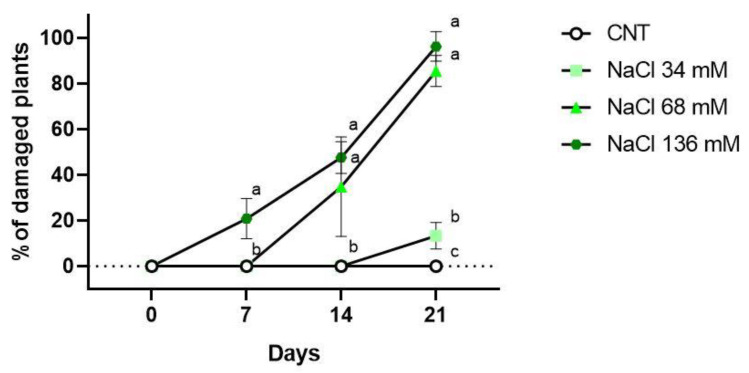
Percentage of plants showing salt injuries in the control (CNT), and 34 , 68, and 136 mM NaCl groups after 7, 14 and 21 days of treatment. Different letters mean statistical difference between the groups (*p* < 0.05).

The data on the percentage of injured plants for each treatment after 7, 14, and 21 days are reported in Figure 3. Necrotic and chlorotic symptoms were not observed after 7 days from the beginning of salt treatments, with the only exception being the highest saline level. The 136 mM NaCl group had 21% of plants with salt injury symptoms, but only on cotyledons. After 2 weeks of treatment, 68 and 136 mM NaCl both showed damage. The 36% of the plants belonging to the 68 mM NaCl group had signs of necrosis and chlorosis on the first true leaves and the cotyledons. In the 136 mM NaCl group, 48% of the samples showed symptoms, and some cotyledons were totally necrotised. The 34 mM NaCl and control groups did not show any symptoms.

At the end of the experiment (21 days), symptoms were observed for all the groups of plants subjected to the various salt treatments, as reported in Figure 3. The 34 mM NaCl group had 13% of plants with symptoms, but there was no statistical difference compared to the control group. However, the 68 and 136 mM NaCl groups showed salt injuries on 86% and 96% of the plant shoots, respectively.

**Figure 4:**
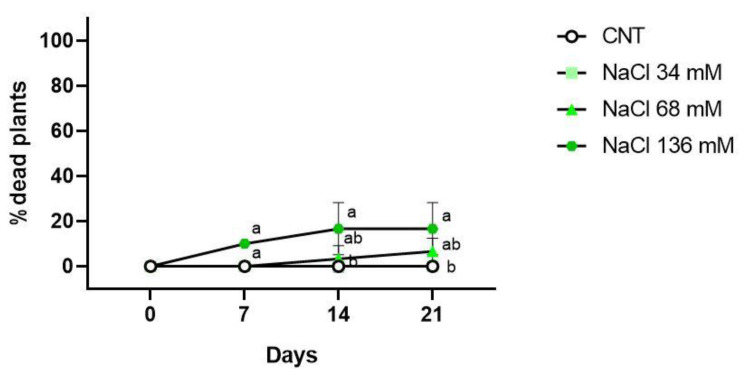
Percentage of dead plants in the control (CNT), and 34, 68 , and 136 mM NaCl groups after 7, 14, and 21 days of treatment. Different letters mean statistical difference between the groups (*p* < 0.05).

Regarding the percentage of dead plants, only the 136 mM NaCl treatment showed dead plants (10%) after 7 days of growth, as indicated in Figure 4. At the end of the second week of treatment, groups showing the occurrence of dead plants were the 68 mM (3%) and 136 mM (17%) NaCl groups. After 21 days from the beginning of the treatments, no dead plants were observed in the control and 34 mM NaCl samples; in the 68 and 136 mM NaCl samples, the percentages of dead plants were 7% and 17%, respectively.

### 2.3. Effect of Salt Stress on Glucosinolate Concentration

**Figure 5:**
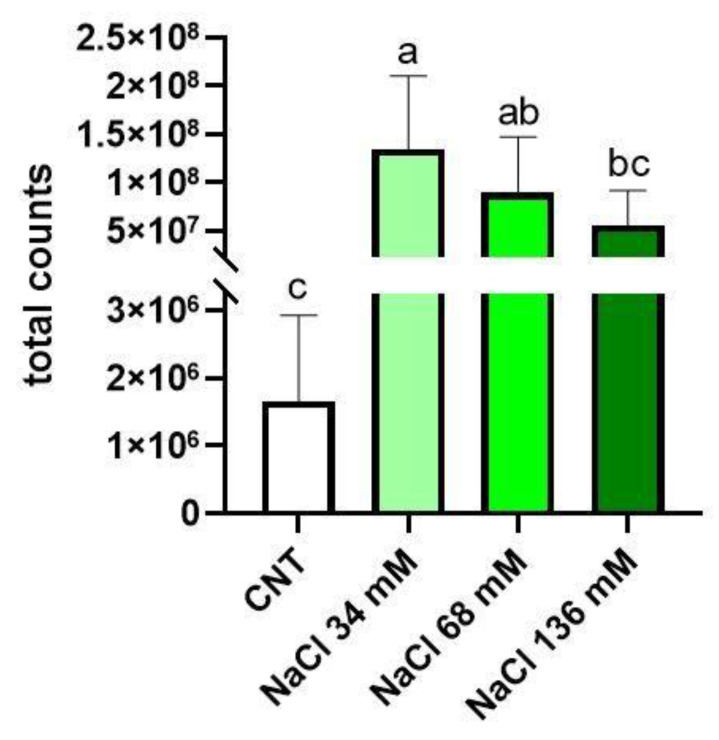
Total glucosinolate concentration in the control group (CNT) compared to the treated groups (36, 68, and 136 mM NaCl). Total counts refer to the sum of the peak areas of glucosinolates in LC–HRMS analysis. Different letters mean statistical difference between the groups (*p* < 0.05).

Figure 5 shows that the highest level of glucosinolates was found in the group treated with 34 mM NaCl in comparison with the control. In the 34 mM NaCl treatment, the glucosinolate level of the samples was similar to that found in the intermediate treated plants (68 mM NaCl), but significantly higher with respect to the control and the 136 mM-treated samples.

**Figure 6:**
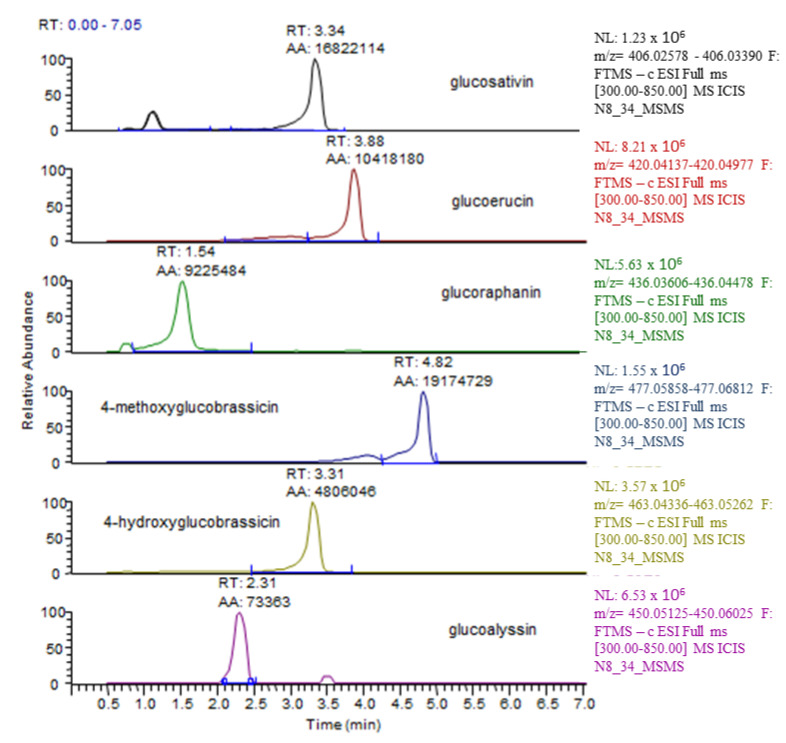
Typical LC–HRMS ion chromatograms detected in *E. sativa* shoots. For each glucosinolate molecule, the *m*/*z* of the [M − H]^−^ was extracted in a 5 ppm mass window.

In *Eruca sativa* shoots, six glucosinolates were recorded with LC–HRMS belonging to two classes: aliphatic (glucosativin, glucoraphanin, glucoalyssin, glucoerucin) and indolic (4-methoxyglucobrassicin and 4-hydroxyglucobrassicin). The LC–HRMS chromatograms of glucosinolates detected in the aerial part are shown in Figure 6. The glucoerucin peak was confirmed via a comparison with an authentic standard, while the remaining five glucosinolates were putatively identified on the basis of experimental accurate mass, tandem MS experiments, and chromatographic behaviour (Figure S1).

**Figure 7:**
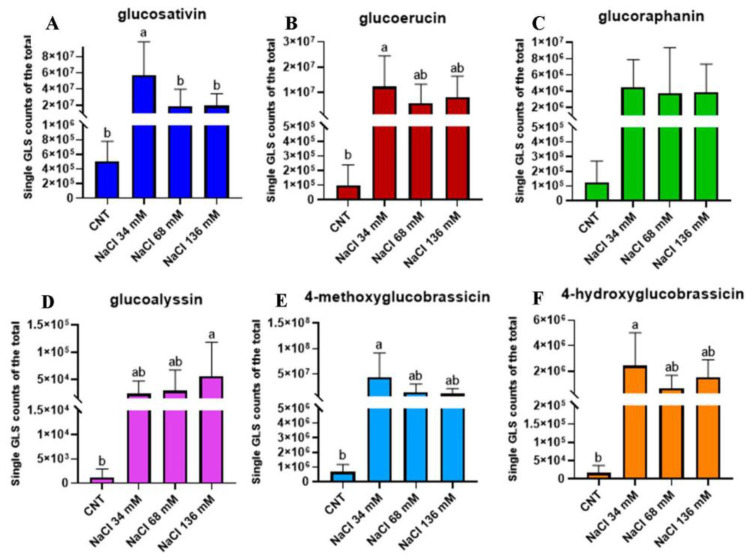
Proportional counts of single GLS: (**A**) glucosativin (dark blue), (**B**) glucoerucin (wine), (**C**) glucoraphanin (green), (**D**) glucoalyssin (violet), (**E**) 4-methoxyglucobrassicin (light blue), and (**F**) 4-hydroxyglucobrassicin (orange) over the total identified with LC–HRMS in the controls compared with the treated groups: 34, 68, and 136 mM NaCl. The percentage was relative to the integration of the peak area and was calculated with respect to the sum of all the peaks. The relative percentages are thus approximate, and the analysis was only a relative quantitation. Different letters mean statistical difference between the groups (*p* < 0.05).

In order to compare the signals of glucosinolates in the different samples, the same ionisation yield was assigned to the five molecules, thus considering the signal intensity (expressed as the area of the chromatographic peak of the respective extracted *m*/*z* value in a 5 part-per-million window) proportional to the amount of the molecule and allowing for a relative comparison of the glucosinolate amount. In general, the amount of aliphatic glucosinolates was higher than that of indole glucosinolates. To implement this analysis, the proportional counts of a single glucosinolate over the total were evaluated. Glucosativin increased in the plants treated with 34 mM NaCl (Figure 7A) in comparison to the control and the other salt treatments. The trends found for glucoerucin (Figure 7B), 4-methoxyglucobrassicin (Figure 7E), and 4-hydroxyglubrassicin (Figure 7F) were similar, and the highest value of counts was recorded for 34 mM NaCl, which was significantly different from the control, but not for the other treatments. The proportional counts recorded for glucoalyssin in (Figure 7D) were significantly higher in the 136 mM NaCl sample, whose value was different from the control, but similar to those of the other saline levels. Figure 7C shows that the glucoraphanin counts were similar in all treatments.

## 3. Discussion

Our results suggest that *E. sativa* could be cultivated at 34 mM salt concentration without any detrimental effect on plant shoot development, thus highlighting its saline adaptation under that level of stress, at least after 21 days. Conversely, the reduction in shoot growth observed in the rocket plants cultivated at 68 and 136 mM NaCl levels probably occurred due to the suppression of leaf expansion and the reduced initiation of new leaves generated by saline stress [11,47,48]. Baby leaf plants demonstrated lower tolerance to salinity in respect to rocket seedlings exposed to 136 mM NaCl, since no impairments in seedling development were observed by Corti et al. [42] at that concentration after 5 days of treatment. Prolonged exposure to 136 mM NaCl concentration may have eventually led to reduced plant development.

Together with the reduction in shoot growth, symptoms of chlorosis and necrosis were observed on the leaves of the plants grown under saline conditions. The symptoms on the leaves generated by salt exposure might have been the result of the detrimental accumulation of Na^+^ and Cl^−^ in the plant [49]. The same toxicity symptoms on leaves were recorded for *Vicia faba* [50], soybean [51], and two rose rootstocks [52] exposed to salinity.

The percentage of the shoot area affected by salt injuries after 21 days of treatment increased with the rising saline level. Considering the European market standard regulation for similar vegetable products, such as lettuce (rocket standards do not exist), the rocket plants cultivated at 34 mM NaCl concentration would be classified as a Class I product and hence still be valuable for market purposes. Conversely, in the 68 mM NaCl group, the high percentage of plants with a wide shoot area affected by salt injuries and plants grown at 136 mM NaCl would be discarded by the market due to the even higher percentage of plant parts damaged by salinity. Hence, neither group would be classified as Class I. Besides the EU regulation, according to the report of Lana and Moita (2019) [13] on consumer choice, vegetable products grown at 68 and 136 mM NaCl would be wasted since consumers tend to discard vegetables showing leaf discoloration or necrosis.

On the other hand, focusing on bioactive compounds, salt stress positively affected the production of glucosinolates in rocket plants. The highest increment was observed at a mild stress level (34 mM), while a lower increase was observed when the levels of salinity were higher (68 and 136 mM). The same trend was recorded by Lopez-Berenguer et al. [53] for broccoli plants subjected to the salt stress of 40 and 80 mM for 15 days. According to Martínez-Ballesta et al. [54], and Sarıkamış and Çakır [55], the increment in total glucosinolates in saline conditions could indicate their involvement in osmotic adjustment, opposing low water potential and helping in coping with the osmotic stress of salt to maintain cell turgor. The reduced level of glucosinolates in the shoots of plants cultivated at the two highest levels of salinity (68 and 136 mM) with respect to the ones grown at the lowest salinity (34 mM) could have been due to salt damage to the membranes of the mesophyll cells. In this way, membrane damage could bring myrosinase in contact with glucosinolates, leading to their hydrolysis and subsequent reduction [19,55]. On the other hand, another explanation for the reduction in total glucosinolates at higher saline levels could have been the inhibition of their biosynthetic pathway at precise salt conditions, since several studies reported that salinity could reduce the metabolic activity of plant cells [19,56]. Since salinity can decrease sulphates because chloride competes with sulphates at the uptake level, the available sulphur could be redistributed to the primary assimilation, thus limiting glucosinolate synthesis [57,58,59].

A previous study, by Kusvuran and Ellialtioglu, on *Eruca sativa* grown under saline conditions showed different results [45]. In particular, this work, on rocket seedlings cultivated in soil with 80 mM NaCl, exhibited a decrease in the total glucosinolate amount, probably due to a substrate-dependent effect on their production. Moreover, plants were cultivated for 41 days, leading to the hypothesis that rocket age may influence glucosinolate production, thus suggesting that baby leaf plants are better suited for glucosinolate enrichment by salt treatment. Focusing on the variation in glucosinolate composition, a higher relative level of the aliphatic compounds was recorded with an increase in salinity. The rise in aliphatic glucosinolates under salt stress conditions was reported in a wild-type and myb28 Arabidopsis mutant [60], *Thellungiella* plants [19], and *Brassica oleracea* [61], whereas a reduction in these compounds was observed in a wild rocket by Cocetta et al. [15]. An increase in the proportions of indole glucosinolates was recorded in *E. sativa* alongside an increase in salinity. An increment in indole glucosinolates under salt stress was also observed by Keling and Zhujun (2010) [62] in pak choi shoots (*Brassica campestris* L. ssp. *Chinensis* var. *communis*).

The major increase in aliphatic glucosinolates with respect to indole glucosinolates observed in *E. sativa* is in agreement with Lopez-Berenguer et al. [53], and Pang et al. [19], who suggested that aliphatic glucosinolates are the most effective in osmotic adjustment and thus as an adaptive mechanism of salt response. The findings of several studies about variation in glucosinolate composition in plants grown under salinity are species-specific, and further investigations are necessary to understand the mechanism of the profile modification of glucosinolates during salt stress conditions [61,63].

Studying glucosinolate variation could be useful in understanding the conditions in which *Brassica* vegetables could be cultivated to enrich their nutraceutical properties [64]. Indeed, a dietary intake of glucosinolates and their derivatives has positive effects, reducing some cancer risk at a number of sites, such as the lungs, stomach, colon, and rectum [65], and reducing mutagenesis, and the toxicity of electrophiles and reactive forms of oxygen [66]. In particular, *Eruca sativa* is used in food and medicine due to its many beneficial features, such as antioxidant, antidiabetic, antimicrobial, anticancer, and anti-inflammatory properties linked to its glucosinolate content [67,68,69]. In this context, a vegetable product enriched in glucosinolates resulting from the cultivation of rocket plants under saline conditions could turn into a food with improved nutraceutical properties. Moreover, a vegetable product that is no longer appealing by consumers due to salt damages, but enriched in glucosinolates, could nevertheless be a source of bioactive compounds in the medical field.

## 4. Materials and Methods

### 4.1. Plant Growth

The seeds of *Eruca sativa* were purchased from the Blumen group S.p.a. and were placed in wrapped filter papers moistened with water for 10 days. Then, seedlings were transferred in hydroponic 50 mL Falcon tubes filled with Hoagland solution [70]. The nutrient solution was renewed every 7 days, and the cultivation lasted 21 days to result in a baby-leaf or microgreen vegetable product [71].

Salt treatments were carried out by adding NaCl to the Hoagland solution to obtain the following salt concentrations: 0.2% (*w*/*v*, 34 mM), 0.4% (*w*/*v*, 68 mM) and 0.8% (*w*/*v*, 136 mM). The salt levels were selected on the basis of a previous study by Corti et al. [42] on *Eruca sativa* grown in saline conditions, highlighting that the seedlings were still able to develop up to 136 mM NaCl.

Seeds and plants were kept in a growth chamber with 10/14 h day/night, a light intensity of 200 µmol m^−2^ s^−1^, a temperature of 24 °C, and humidity of 54%.

Ten plants were cultivated for each treatment, and the experiment was conducted in triplicate. The following analysis was performed on the aerial parts, since the shoots represent the commercially exploited plant parts.

### 4.2. Shoot Coverage Area

Pictures of the samples were taken at 7, 14, and 21 days from the beginning of the experiment using a Canon PowerShot S X100IS camera. The photos were used to measure the surface covered by the shoots of each plant for each treatment. The images were analysed with Fiji 2.3.1 software [72].

### 4.3. Shoot Injury Observation

Necrotic and chlorotic symptoms were checked out every 7 days until the end of the experiment (21 days). The damage extent is reported as the percentage of the shoot area affected by both necrosis and chlorosis. Furthermore, the percentages of plants showing shoot injuries and of dead plants were determined at 7, 14, and 21 days from the beginning of the experiment for each treatment.

### 4.4. Glucosinolate Extraction

Glucosinolates were extracted on the basis of the protocol proposed by Cataldi et al. [73]. The shoots of 21-day-old plants were cut, frozen in liquid nitrogen, and stored at −80 °C. Then, the samples were powdered in liquid nitrogen using a mortar and pestle. Then, 100 mg of powder was suspended in a 1 mL solution composed of 700 µL of CH_3_OH and 300 µL of H_2_O, and incubated at 75 °C for 10 min. The samples were centrifuged (15 min at 14,000× *g*), and the supernatants were collected. The extraction procedure was repeated on solid residue, and the new supernatants were added to the ones collected before. The samples were concentrated with evaporation to dryness at 40 °C under a vacuum in a rotary evaporator, and redissolved in 1 mL of a CH_3_OH/H_2_O 7:3 (*v*/*v*) solution. The extracts were filtered using a single-use 0.45 µm Nylon filter and stored in a screw-cap vial.

In light of the limited amount of the samples obtained from the studied *Eruca sativa* plants, a sample of salad rocket purchased from a local supermarket was used as a quality-control (QC) sample, visually verifying that only salad-rocket leaves were present. Several aliquots of this QC were processed in parallel with the actual samples, and used to verify the instrumental stability and reproducibility of measurements during the batch sequence and between batches in different days. All the samples were processed and analysed over the course of 2 weeks. A QC extract was also used to assess the stability of the glucosinolates in the autosampler plate, injecting it in different days: a variation of less than 10% in area counts was recorded for all the monitored glucosinolates after 5 days. Using the QC sample and some replicated injections of an extract of the actual samples, no analytical batch effect was recorded, and comparable results were obtained for each glucosinolate, and observing area counts between 85 and 115% of the calculated average area counts of each glucosinolate in the QC and actual samples. For only glucoalyssin and 4-hydroxyglucobrassicin in the control samples, ± 25% was observed due to the poor signal intensity.

### 4.5. Glucosinolate Analysis

The extracts were analysed with LC–HRMS using a Thermo Scientific (Bremen, Germany) instrument composed of an Ultimate 3000 HPLC coupled to a LTQ Orbitrap mass spectrometer via an IonMax ESI interface. The column was a Kinetex EVO C18 (Phenomenex, Torrance, CA, USA), 100 × 2.1 mm, 5 µm, operating at 250 µL/min and thermostated at 35 °C. The eluents were water (Phase A) and acetonitrile (Phase B), both containing 0.1% formic acid (FA). The injection volume was 10 µL. The HPLC injector sample plate was thermostated at 6 (±2) °C. The gradient elution program was as follows: 0 min, 2% B; a linear gradient to 65% B in 8.8 min; a linear gradient to 97% B in 2 min, left for 2 min; the initial conditions were then restored and left to equilibrate for 10 min. After 0.5 min from the sample injection, the elution from the column was directly transferred to the ESI interface operating with the following settings: ESI voltage, 4.8 kV; capillary temperature, 280 °C; capillary voltage, −24 V’; tube lens, −63 V. Sheath, auxiliary, and sweep gases were at 29, 5 and 5 arbitrary units, respectively. High-resolution mass spectra were recorded in negative ion mode at 60,000 resolution (at 400 *m*/*z*), from 300 to 850 *m*/*z*. The orbitrap analyser was calibrated immediately before batch analysis using the calibration mixture suggested by Thermo.

Each analytical sequence included blank samples (CH_3_OH/H_2_O 7:3, *v*/*v*), QC extracts, a standard glucoerucin solution at different concentrations (for instrumental linearity check and stability of mass accuracy), and actual salad-rocket extracts; the sequence started with at least two blank samples and two QC injections. The following injections were randomised, and at the end of the sequence, two injections of the QC samples and two blank samples were performed again. Each actual sample was injected three times, and the area count reproducibility was checked; special attention was paid to samples injected after the other samples with high glucosinolate signals to verify that the potential memory effect was null or negligible.

Instrument linearity was checked by injecting scalar amounts of a glucoerucin standard solution: the amount of glucoerucin injected into the LC column ranged from 1 to 1000 ng. We did not stress linearity towards amounts lower than 1 ng to better estimate the areas of glucosinolates present at lower levels, having already observed a clear difference in the signals between control and salt-stressed samples in some preliminary experiments.

A linear relationship between the injected amount and area counts for the deprotonated ion of glucoerucin (extracted in a 5 ppm mass window) was recorded; the response linearity was checked at the beginning and at the end of each analytical batch, obtaining r^2^ values ranging from 0.989 to 0.991. A similar relationship was observed for the actual samples in which higher area counts were recorded for the most intense glucosinolates signals (glucosativin, glucoerucin, glucoraphanin and 4-methoxyglucobrassicin). We analysed the same extract as it was, and after 1-to-2 and 1-to-4 dilution with 0.1% FA: the linear relationship among the three recorded area counts was comparable to that observed for the glucoerucin standard, even if the slope was not identical due to the reduced matrix effect after dilution.

The measured linearity was well-suited to the purpose of our study, which was to estimate the relative fold change in glucosinolate content under the different tested conditions.

During method development, the matrix effect was evaluated according to Bonfiglio et al. [74] with the postcolumn infusion of a standard 250 ng/μL solution of glucoerucin at 5 μL/min using a T-connection between the column exit and the ESI interface. The deprotonated ion of glucoerucin was monitored in the elution time window of the glucosinolates, and its intensity was compared after the analysis of a solvent and a salad-rocket extract. The QC and actual samples were tested, showing almost identical ion suppression, around 15–20% of the glucoerucin signal, in agreement with other published studies. This evaluation was not possible for glucoerucin due to its natural presence in the extract, but similar suppression was expected due to the stability of the signal immediately before and after the chromatographic peak of glucoerucin.

### 4.6. Statistical Analysis

The data were processed with GraphPad Prism (version 8.0.0 for Windows, GraphPad Software, San Diego, CA, USA, www.graphpad.com, accessed on 15 November 2022) using one-way ANOVA followed by Tukey’s multiple-comparisons test.

## 5. Conclusions

Rocket plants grown in saline conditions resulted in products with an increased glucosinolate level. However, two distinct products could be characterised when taking into account both glucosinolates and the functional traits influencing the aesthetic aspect of *E. sativa* plants. The plants cultivated at 68 and 136 mM NaCl concentrations would be considered waste products following the EU’s Common Market Organisation standards, but due to their high level of glucosinolates, their use as a source of these bioactive compounds should be taken into account. On the other hand, *E. sativa* plants grown under 34 mM NaCl concentration resulted in a win–win situation. Indeed, they can be considered Class I sealable microgreen and baby-leaf vegetables with an increased level of glucosinolates, thus resulting in an improved nutraceutical product.

## Data Availability

Not applicable.

## Supplementary Materials

The following supporting information can be downloaded at: https://www.mdpi.com/article/10.3390/ijms24065510/s1.

## References

[1] Munns R., Tester M. (2008). Mechanisms of salinity tolerance. Annu. Rev. Plant Biol..

[2] Shahbaz M., Ashraf M. (2013). Improving salinity tolerance in cereals. Crit. Rev. Plant Sci..

[3] Jamil A., Riaz S., Ashraf M., Foolad M.R. (2011). Gene expression profiling of plants under salt stress. Crit. Rev. Plant Sci..

[4] Shrivastava P., Kumar R. (2015). Soil salinity: A serious environmental issue and plant growth promoting bacteria as one of the tools for its alleviation. Saudi J. Bio. Sci..

[5] Ivanova K., Dimitrova V., Georgieva T., Markovska Y. (2015). Effect of soil salinity on growth, gas exchange and antioxidant defence of two Paulownia lines. Gen. Plant Physiol..

[6] Parida A.K., Das A.B. (2005). Salt tolerance and salinity effects on plants: A review. Ecot. Environ. Saf..

[7] Paul D., Lade H. (2014). Plant-growth-promoting rhizobacteria to improve crop growth in saline soils: A review. Agro. Sust. Dev..

[8] Tavakkoli E., Fatehi F., Coventry S., Rengasamy P., McDonald G.K. (2011). Additive effects of Na^+^ and Cl^−^ ions on barley growth under salinity stress. J. Exp. Bot..

[9] Arbona V., Marco A.J., Iglesias D.J., López-Climent M.F., Talon M., Gómez-Cadenas A. (2005). Carbohydrate depletion in roots and leaves of salt-stressed potted *Citrus clementina* L. Plant Growth Regul..

[10] Cramer G.R., Nowak R.S. (1992). Supplemental manganese improves the relative growth, net assimilation and photosynthetic rates of salt-stressed barley. Phys. Plant..

[11] Acosta-Motos J.R., Ortuño M.F., Bernal-Vicente A., Diaz-Vivancos P., Sanchez-Blanco M.J., Hernandez J.A. (2017). Plant responses to salt stress: Adaptive mechanisms. Agronomy.

[12] European Commission (2011). Commission Implementing Regulation (EU) No 543/2011: Laying down detailed rules in respect of the fruit and vegetables and processed fruit and vegetables sectors. Off. J. Eur. Union.

[13] Lana M.M., Moita A.W. (2019). Visual quality and waste of fresh vegetables and herbs in a typical retail market in Brazil. Hortic. Bras..

[14] Porter S.D., Reay D.S., Bomberg E., Higgins P. (2018). Avoidable food losses and associated production-phase greenhouse gas emissions arising from application of cosmetic standards to fresh fruit and vegetables in Europe and the UK. J. Clean. Prod..

[15] Cocetta G., Mishra S., Raffaelli A., Ferrante A. (2018). Effect of heat root stress and high salinity on glucosinolates metabolism in wild rocket. J. Plant Physiol..

[16] Orsini F., Maggio A., Rouphael Y., De Pascale S. (2016). “Physiological quality” of organically grown vegetables. Sci. Hortic..

[17] Moya C., Oyanedel E., Verdugo G., Fernanda Flores M., Urrestarazu M., Alvaro J.E. (2017). Increased electrical conductivity in nutrient solution management enhances dietary and organoleptic qualities in soilless culture tomato. HortScience.

[18] Alessio P., Mosti S., Tani G., Falco P.D., Lazzeri L., Bandara N.L. (2010). Ultrastructural aspects of the embryo and different endosperm compartments, in *Eruca sativa* Hill cv. Nemat (*Brassicaceae*) during Heart and Torpedo stages. Caryologia.

[19] Pang Q., Guo J., Chen S., Chen Y., Zhang L., Fei M., Jin S., Li M., Wang Y., Yan X. (2012). Effect of salt treatment on the glucosinolate-myrosinase system in *Thellungiella salsuginea*. Plant Soil.

[20] Guo L., Yang R., Wang Z., Guo Q., Gu Z. (2014). Effect of NaCl stress on health-promoting compounds and antioxidant activity in the sprouts of three broccoli cultivars. Int. J. Food Sci. Nutr..

[21] Yuan G., Wang X., Guo R., Wang Q. (2010). Effect of salt stress on phenolic compounds, glucosinolates, myrosinase and antioxidant activity in radish sprouts. Food Chem..

[22] Chen Y., Wang Y., Huang J., Zheng C., Cai C., Wang Q., Wu C.A. (2017). Salt and methyl jasmonate aggravate growth inhibition and senescence in Arabidopsis seedlings via the JA signaling pathway. Plant Sci..

[23] Sarker U., Oba S. (2018). Drought stress enhances nutritional and bioactive compounds, phenolic acids and antioxidant capacity of *Amaranthus* leafy vegetable. BMC Plant Biol..

[24] Halkier B.A., Gershenzon J. (2006). Biology and biochemistry of glucosinolates. Ann. Rev. Plant Biol..

[25] Wittstock U., Halkier B.A. (2002). Glucosinolate research in the Arabidopsis era. Trends Plant Sci..

[26] Almuhayawi M.S., AbdElgawad H., Al Jaouni S.K., Selim S., Hassan A.H., Khamis G. (2020). Elevated CO_2_ improves glucosinolate metabolism and stimulates anticancer and anti-inflammatory properties of broccoli sprouts. Food Chem..

[27] Steinbrecher A., Nimptsch K., Hüsing A., Rohrmann S., Linseisen J. (2009). Dietary glucosinolate intake and risk of prostate cancer in the EPIC-Heidelberg cohort study. Int. J. Cancer.

[28] London S.J., Yuan J.M., Chung F.L., Gao Y.T., Coetzee G.A., Ross R.K., Mimi C.Y. (2000). Isothiocyanates, glutathione S-transferase M1 and T1 polymorphisms, and lung-cancer risk: A prospective study of men in Shanghai, China. Lancet.

[29] Poppel G.V., Verhoeven D.T., Verhagen H., Goldbohm R.A. (1999). Brassica vegetables and cancer prevention. Adv. Nutr. Cancer.

[30] Seow A., Yuan J.M., Sun C.L., Van Den Berg D., Lee H.P., Yu M.C. (2002). Dietary isothiocyanates, glutathione S-transferase polymorphisms and colorectal cancer risk in the Singapore Chinese Health Study. Carcinogenesis.

[31] Bhattacharya A., Li Y., Wade K.L., Paonessa J.D., Fahey J.W., Zhang Y. (2010). Allyl isothiocyanate-rich mustard seed powder inhibits bladder cancer growth and muscle invasion. Carcinogenesis.

[32] Fowke J.H., Chung F.L., Jin F., Qi D., Cai Q., Conaway C., Cheng J.-R., Shu X.-O., Gao Y.-T., Zheng W. (2003). Urinary isothiocyanate levels, brassica, and human breast cancer. Cancer Res..

[33] Rouphael Y., Kyriacou M.C. (2018). Enhancing quality of fresh vegetables through salinity eustress and biofortification applications facilitated by soilless cultivation. Front. Plant Sci..

[34] Martínez-Sánchez A., Marín A., Llorach R., Ferreres F., Gil M.I. (2006). Controlled atmosphere preserves quality and phytonutrients in wild rocket (*Diplotaxis tenuifolia*). Postharvest Biol. Technol..

[35] Pasini F., Verardo V., Cerretani L., Caboni M.F., D’Antuono L.F. (2011). Rocket salad (*Diplotaxis* and *Eruca* spp.) sensory analysis and relation with glucosinolate and phenolic content. J. Sci. Food Agric..

[36] Awadelkareem A.M., Al-Shammari E., Elkhalifa A.O., Adnan M., Siddiqui A.J., Mahmood D., Azad Z.R.A.A., Patel M., Mehmood K., Danciu C. (2022). Anti-Adhesion and Antibiofilm Activity of *Eruca sativa* Miller Extract Targeting Cell Adhesion Proteins of Food-Borne Bacteria as a Potential Mechanism: Combined In Vitro-In Silico Approach. Plants.

[37] Awadelkareem A.M., Al-Shammari E., Elkhalifa A.O., Adnan M., Siddiqui A.J., Patel M., Khan M.I., Mehmood K., Ashfaq F., Badraoui R. (2022). Biosynthesized Silver Nanoparticles from *Eruca sativa* Miller Leaf Extract Exhibits Antibacterial, Antioxidant, Anti-Quorum-Sensing, Antibiofilm, and Anti-Metastatic Activities. Antibiotics.

[38] Awadelkareem A.M., Al-Shammari E., Elkhalifa A.E.O., Adnan M., Siddiqui A.J., Snoussi M., Khan M.I., Azad Z.R.A.A., Patel M., Ashraf S.A. (2022). Phytochemical and In Silico ADME/Tox Analysis of *Eruca sativa* Extract with Antioxidant, Antibacterial and Anticancer Potential against Caco-2 and HCT-116 Colorectal Carcinoma Cell Lines. Molecules.

[39] Bozokalfa K.M., Eşiyok D., Yağmur B. (2011). Use of multivariate analysis in mineral accumulation of rocket (*Eruca sativa*) accessions. Genetika.

[40] Tomaz A., Palma P., Fialho S., Lima A., Alvarenga P., Potes M., João Costa M., Salgado R. (2020). Risk assessment of irrigation-related soil salinization and sodification in Mediterranean areas. Water.

[41] Solé J., Samsó R., García-Ladona E., García-Olivares A., Ballabrera Poy J., Madurell T., Turiel A., Osychenko O., Álvarez D., Bardi U. (2020). Modelling the renewable transition: Scenarios and pathways for a decarbonized future using pymedeas, a new open-source energy systems model. Renew. Sustain. Energy Rev..

[42] Corti E., Falsini S., Schiff S., Tani C., Gonnelli C., Papini A. (2023). Saline Stress Impairs Lipid Storage Mobilization during Germination in *Eruca sativa*. Plants.

[43] Bakhshandeh E., Pirdashti H., Vahabinia F., Gholamhossieni M. (2020). Quantification of the effect of environmental factors on seed germination and seedling growth of Eruca (*Eruca sativa*) using mathematical models. J. Plant Growth Regul..

[44] Fallahi H.R., Fadaeian G., Gholami M., Daneshkhah O., Hosseini F.S., Aghhavani-Shajari M., Samadzadeh A. (2015). Germination response of grasspea (*Lathyrus Sativus* L/) and arugula (*Eruca Sativa* L.) to osmotic and salinity stresses. Plant Breed. Seed Sci..

[45] Kusvuran S., Ellialtioglu S.S. (2021). Assessment of different organic matters on antioxidative enzyme activities and nutritional components under salt stress in salad rocket (*Eruca sativa*). JAPS J. Anim. Plant Sci..

[46] Petretto G.L., Urgeghe P.P., Massa D., Melito S. (2019). Effect of salinity (NaCl) on plant growth, nutrient content, and glucosinolate hydrolysis products trends in rocket genotypes. Plant Physiol. Biochem..

[47] Akram M., Ashraf M.Y., Ahmad R., Rafiq M., Ahmad I., Iqbal J. (2010). Allometry and yield components of maize (*Zea mays* L.) hybrids to various potassium levels under saline conditions. Arch. Biol. Sci..

[48] Qu C., Liu C., Gong X., Li C., Hong M., Wang L., Hong F. (2012). Impairment of maize seedling photosynthesis caused by a combination of potassium deficiency and salt stress. Env. Exp. Bot..

[49] Munns R. (2009). Strategies for crop improvement in saline soils. Salin. Water Stress.

[50] Slabu C., Zörb C., Steffens D., Schubert S. (2009). Is salt stress of faba bean (*Vicia faba*) caused by Na^+^ or Cl^−^toxicity?. J. Plant Nutr. Soil Sci..

[51] Lenis J.M., Ellersieck M., Blevins D.G., Sleper D.A., Nguyen H.T., Dunn D., Lee J.D., Shannon J.G. (2011). Differences in ion accumulation and salt tolerance among Glycine accessions. J. Agron. Crop Sci..

[52] Wahome P.K., Jesch H.H., Grittner I. (2001). Mechanisms of salt stress tolerance in two rose rootstocks: *Rosa chinensis* ‘Major’and *R. rubiginosa*. Sci. Hortic..

[53] Lopez-Berenguer C., Martínez-Ballesta M.D.C., Moreno D.A., Carvajal M., Garcia-Viguera C. (2009). Growing hardier crops for better health: Salinity tolerance and the nutritional value of broccoli. J. Agric. Food Chem..

[54] Martínez-Ballesta M., Moreno D.A., Carvajal M. (2013). The physiological importance of glucosinolates on plant response to abiotic stress in Brassica. Int. J. Mol. Sci..

[55] Sarikamiş G., Cakir A. (2017). Influence of salinity on aliphatic and indole glucosinolates in broccoli (*Brassica oleracea* var. italica). Appl. Ecol. Environ. Res..

[56] Cicek N., Çakirlar H. (2002). The effect of salinity on some physiological parameters in two maize cultivars. Bulg. J. Plant Physiol..

[57] Koprivova A., North K.A., Kopriva S. (2008). Complex signaling network in regulation of adenosine 5′-phosphosulfate reductase by salt stress in Arabidopsis roots. Plant Physiol..

[58] Reich M., Aghajanzadeh T., Helm J., Parmar S., Hawkesford M.J., De Kok L.J. (2016). Chloride and sulfate salinity differently affect biomass, mineral nutrient composition and expression of sulfate transport and assimilation genes in *Brassica rapa*. Plant Soil.

[59] Aghajanzadeh T.A., Reich M., Kopriva S., De Kok L.J. (2018). Impact of chloride (NaCl, KCl) and sulphate (Na_2_SO_4_, K_2_SO_4_) salinity on glucosinolate metabolism in *Brassica rapa*. J. Agro. Crop. Sci..

[60] Martinez-Ballesta M.D.C., Carvajal M. (2015). Myrosinase in Brassicaceae: The most important issue for glucosinolate turnover and food quality. Phytochem. Rev..

[61] Martínez-Ballesta M.D.C., Muries B., Moreno D.Á., Dominguez-Perles R., García-Viguera C., Carvajal M. (2014). Involvement of a glucosinolate (sinigrin) in the regulation of water transport in *Brassica oleracea* grown under salt stress. Physiol. Plant..

[62] Keling H., Zhujun Z. (2010). Effects of different concentrations of sodium chloride on plant growth and glucosinolate content and composition in pakchoi. Afr. J. Biotechnol..

[63] Sytar O., Mbarki S., Zivcak M., Brestic M. (2018). The involvement of different secondary metabolites in salinity tolerance of crops. Salinity Responses and Tolerance in Plants.

[64] Moreno D.A., Carvajal M., López-Berenguer C., García-Viguera C. (2006). Chemical and biological characterisation of nutraceutical compounds of broccoli. J. Pharm. Biomed. Anal..

[65] Prakash D., Gupta C. (2012). Glucosinolates: The phytochemicals of nutraceutical importance. J. Compl. Integr. Med..

[66] Prakash D., Gupta C. (2019). Phytopharmaceutical applications of nutraceutical and functional foods. Complementary and Alternative Medicine: Breakthroughs in Research and Practice.

[67] Gulfraz M., Sadiq A., Tariq H., Imran M., Qureshi R. (2011). Phytochemical analysis and antibacterial activity of Eruca sativa seed. Pak.J. Bot..

[68] Di Gioia F., Avato P., Serio F., Argentieri M.P. (2018). Glucosinolate profile of Eruca sativa, Diplotaxis tenuifolia and Diplotaxis erucoides grown in soil and soilless systems. J. Food Compos. Anal..

[69] Alqasoumi S., Al-Sohaibani M., Al-Howiriny T., Al-Yahya M., Rafatullah S. (2009). Rocket “*Eruca sativa*”: A salad herb with potential gastric anti-ulcer activity. World J. Gastroenterol..

[70] Hoagland D.R., Arnon D.I. (1950). The Water-Culture Method for Growing Plants without Soil.

[71] Di Gioia F., Renna M., Santamaria P. (2017). Sprouts, microgreens and “baby leaf” vegetables. Minimally Processed Refrigerated Fruits and Vegetables.

[72] Schindelin J., Arganda-Carreras I., Frise E., Kaynig V., Longair M., Pietzsch T., Preibisch S., Rueden C., Saalfeld S., Schmid B. (2012). Fiji: An open-source platform for biological-image analysis. Nat. Methods.

[73] Cataldi T.R., Rubino A., Lelario F., Bufo S.A. (2007). Naturally occurring glucosinolates in plant extracts of rocket salad (*Eruca sativa* L.) identified by liquid chromatography coupled with negative ion electrospray ionization and quadrupole ion-trap mass spectrometry. Rapid Commun. Mass Spectrom. Int. J. Devoted Rapid Dissem. Up-Minute Res. Mass Spectrom..

[74] Bonfiglio R., King R.C., Olah T.V., Merkle K. (1999). The effects of sample preparation methods on the variability of the electrospray ionization response for model drug compounds. Rapid Commun. Mass Spectrom..

