# Supplementary material for: Salt-Affected Rocket Plants as a Possible Source of Glucosinolates
Source: Int J Mol Sci. 2023 Mar 14;24(6):5510. doi: 10.3390/ijms24065510 (PMC10056271; doi:10.3390/ijms24065510)
Supplement: Supplementary file 1 [file ijms-24-05510-s001.zip › ijms-2203530-supplementary.pdf]

Supplementay information

Salt-affected rocket plants as a possible source of glucosinolates

Emilio Corti[1], Sara Falsini[1*], Cristina Gonnelli[1], Giuseppe Pieraccini[2], Besiana Nako[1], Alessio Papini[1,3]

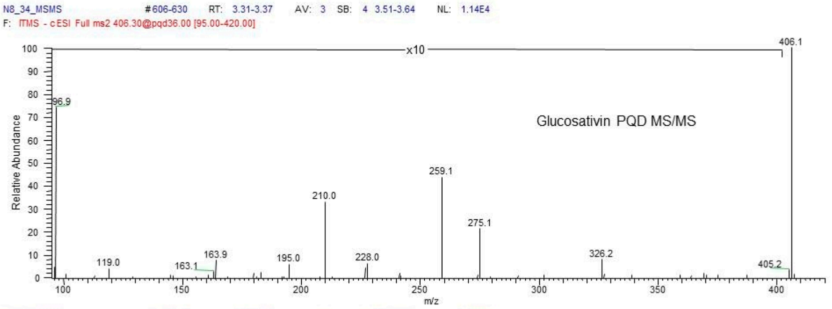

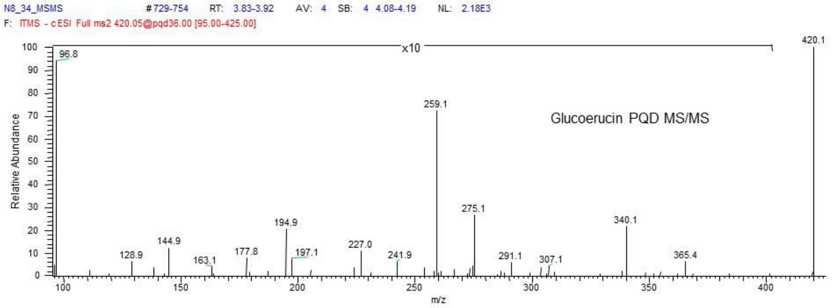

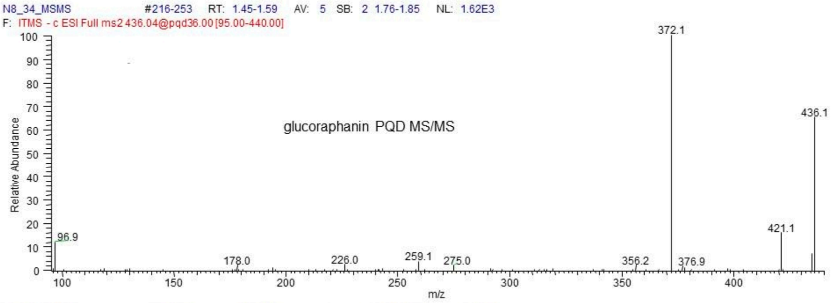

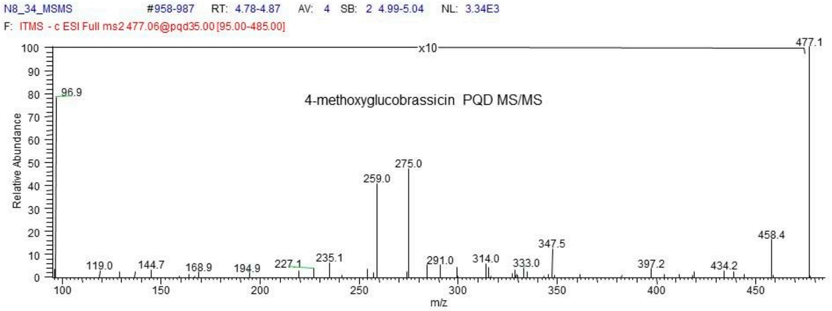

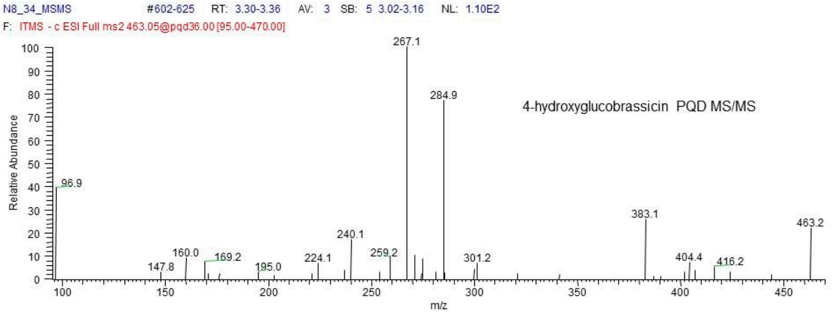

Figure S1: MS/MS spectra of glucosativin, glucoerucin, glucoraphanin, 4-methoxyglucobrassicin and 4 –hydroxyglucobrassicin obtained by Pulsed Q Collision Induced Dissociation (PQD) in the linear quadrupole ion trap (LTQ) analyzer, obtained from the analysis of the rocket extract in Fig. 6.
